# Constructing N‐Containing Poly(*p*‐Phenylene) (PPP) Films Through A Cathodic‐Dehalogenation Polymerization Method

**DOI:** 10.1002/smtd.202400185

**Published:** 2024-04-15

**Authors:** Xiang Wang, Lei Zhang, Jinghang Wu, Miaomiao Xue, Qianfeng Gu, Junlei Qi, Fangyuan Kang, Qiyuan He, Xiaoyan Zhong, Qichun Zhang

**Affiliations:** ^1^ Department of Materials Science and Engineering City University of Hong Kong Kowloon Hong Kong SAR 999077 P. R. China; ^2^ City University of Hong Kong Matter Science Research Institute (Futian, Shenzhen) Shenzhen 518048 P. R. China; ^3^ Nanomanufacturing Laboratory (NML) City University of Hong Kong Shenzhen Research Institute Shenzhen 518057 P. R. China; ^4^ Department of Chemistry Center of Super‐Diamond and Advanced Films (COSDAF) Hong Kong Institute for Clean Energy (HKICE) City University of Hong Kong Kowloon Hong Kong SAR 999077 P. R. China

**Keywords:** cathodic polymerization, electrochemical dehalogenation, electrochemical hydrogen evolution, films, N‐containing poly(*p*‐phenylene) (PPP)

## Abstract

Developing the films of N‐containing unsubstituted poly(*p*‐phenylene) (PPP) films for diverse applications is significant and highly desirable because the replacement of sp^2^ C atoms with sp^2^ N atoms will bring novel properties to the as‐prepared polymers. In this research, an electrochemical‐dehalogenation polymerization strategy is employed to construct two N‐containing PPP films under constant potentials, where 2,5‐diiodopyridine (DIPy) and 2,5‐dibromopyrazine (DBPz) are used as starting agents. The corresponding polymers are named **CityU‐23** (for polypyridine) and **CityU‐24** (for polypyrazine). Moreover, it is found that both polymers can form films in situ on different conductive substrates (i.e., silicon, gold, ITO, and nickel), satisfying potential device fabrication. Furthermore, the as‐obtained thin films of **CityU‐23** and **CityU‐24** exhibit good performance of alkaline hydrogen evolution reaction with the overpotential of 212.8 and 180.7 mV and the Tafel slope of 157.0 and 122.4 mV dec^−1^, respectively.

## Graphical abstract

The cathodic‐dehalogenation strategy is modified to simultaneously prepare two N‐containing poly(*p*‐phenylene)s (**CityU‐23** from 2,5‐diiodopyridine (DIPy) and **CityU‐24** from 2,5‐dibromopyrazine (DBPz)) and fabricate their corresponding thin films. Both polymer films display good alkaline HER performance, where the efficiency increases with the number of the introduced sp^2^ N atoms.

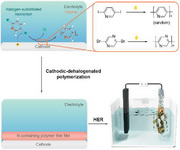

## 1 Introduction

Conjugated polymers have been demonstrated to show a wide range of applications, including transistors,^[^ ^1^ ^]^ energy storage,^[^ ^2^ ^]^ thermoelectric devices,^[^ ^3^ ^]^ sensors,^[^ ^4^ ^]^ catalysis,^[^ ^5^, ^6^ ^]^ etc., due to their tunable physical and chemical properties. Constructing C─C bonds between aryl repeating units is significant among all kinds of polymerization.^[^ ^7^ ^]^ Till now, many synthetic methods, such as the Ullmann reaction,^[^ ^8^ ^]^ Grignard reaction, Suzuki reaction,^[^ ^9^ ^]^ Stille and Negishi coupling,^[^ ^10^, ^11^, ^12^ ^]^ etc., have been employed to build the C─C bonds.^[^ ^13^ ^]^ However, their unsatisfied reaction conditions/by‐products (i.e., organo‐noble‐metallic catalyst (i.e., the organopalladium catalyst for Suzuki), toxic organometallic byproducts (i.e., trialkylstannanes for Stille), or the impurities caused by the remains of metallic nanoparticles which would be detrimental to electronic applications (though some would improve the performance)), are inevitable during the polymerization.^[^ ^13^ ^]^ Therefore, developing novel methods to achieve clean C─C‐based polymerization under mild conditions is essential. Moreover, although various film fabrication methods, such as the on‐surface polymerization,^[^ ^14^ ^]^ spin‐coating,^[^ ^15^ ^]^ interfacial reaction,^[^ ^16^, ^17^ ^]^ Langmuir–Blodgett film,^[^ ^18^ ^]^ and so on, have been reported, developing an integrated method to simultaneously conduct polymerization and film fabrication on diverse substrates under mild condition is necessary to meet the demands of numerous devices and applications.^[^ ^19^, ^20^ ^]^ However, such a manufacturing process is difficult due to the speed control of polymerization and the poor solubility of the as‐obtained unsubstituted conjugated polymers.^[^ ^7^ ^]^

Since many organic reactions involve the transfer and recombination of electrons, using electrons as the catalyst to perform polymerization should be a solution to address the issues mentioned above.^[^ ^21^ ^]^ The electrochemical oxidative polymerizations of thiophene, furan, and pyrrole are good examples.^[^ ^22^, ^23^, ^24^, ^25^ ^]^ Furthermore, the electrochemical technique has been widely considered one of the precisely controllable methods with potential industrial applications through two strategies to prepare thin films: electrochemical deposition (ECD) and electrophoretic deposition (EPD).^[^ ^7^, ^20^ ^]^ Therefore, the electrochemical methods can be considered, to some extent, as an integration of organic polymer synthesis and corresponding film fabrication. Based on these factors, our group has successfully applied a cathodic‐dehalogenation polymerization strategy in simultaneous synthesis and thin film fabrication of benzene/polycyclic aromatic hydrocarbon‐based conjugated polymers.^[^ ^7^ ^]^ This success strongly inspires us to switch to other substrates, especially sp^2^‐N‐containing polycyclic aromatic hydrocarbons, because introducing heteroatoms (N, S, O, P, etc.) into the aromatic ring could bring more novel properties.^[^ ^26^, ^27^, ^28^, ^29^ ^]^ For instance, the Cu(I)─N coordination polymer (NNU‐33(S)) shows excellent performance of catalytic behaviors and high selectivity of the product CH_4_.^[^ ^30^ ^]^ Covalent triazine frameworks exhibit good photocatalytic hydrogen evolution reaction (HER) performance with the co‐catalyst – Pt nanoparticles.^[^ ^31^ ^]^ The metal‐free triazine‐based and porphyrin‐based covalent organic frameworks display potentials in electrochemical HER.^[^ ^32^, ^33^ ^]^ However, till now, the realization of C─C coupling between the heterocyclic aromatic compounds in literature cannot bypass the involvement of organometallic catalysts, even in the electrochemical‐assistant coupling reaction, and more frustratedly, they cannot directly form thin films.^[^ ^7^, ^34^, ^35^, ^36^ ^]^ This gap strongly inspires us to perform this type of research and simultaneously realize polymerization and thin film fabrication through the cathodic‐dehalogenation C─C coupling between the heterocyclic aromatic compounds.

Herein, through the modification of the previous cathodic‐dehalogenation polymerization strategy,^[^ ^7^ ^]^ we can extend the scope of this strategy to the polymerization of N‐containing benzene (i.e., 2,5‐diiodopyridine (DIPy) and 2,5‐dibromopyrazine (DBPz)), and successfully fabricated corresponding thin films on different conductive substrates (i.e., silicon, gold, ITO, and nickel). The corresponding polymers have been named **CityU‐23** (for poly(2,5‐pyridine) from 2,5‐diiodopyridine) and **CityU‐24** (for poly(2,5‐pyrazine) from 2,5‐dibromopyrazine). Moreover, the addition of an appropriate amount of Ph_3_P could decrease the required reduction potential and promote polymerization. Remarkably, since the introduction of active sites – sp^2^ N into the aromatic rings, the as‐obtained metal‐free thin films of **CityU‐23** and **CityU‐24** show good HER catalytic performance in alkaline HER compared with the PPP thin film. The overpotential of **CityU‐23** and **CityU‐24** in alkaline HER are 212.8 and 180.7 mV, respectively. The Tafel slopes of **CityU‐23** and **CityU‐24** are 157.0 and 122.4 mV dec^−1^, respectively. Although **CityU‐24** shows better HER catalysis than **CityU‐23**, probably due to more active sites (sp^2^ N) in the aromatic rings, **CityU‐23** is stable compared with **CityU‐24** during the electrocatalytic process.

## 2 Results and Discussion

### 2.1 The Synthesis and Characterization

**Scheme 1:**
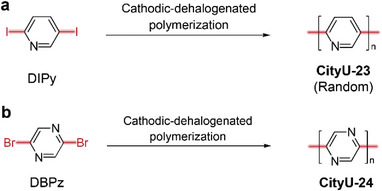
The schematic of preparation of CityU‐23 and CityU‐24.

**Figure 1:**
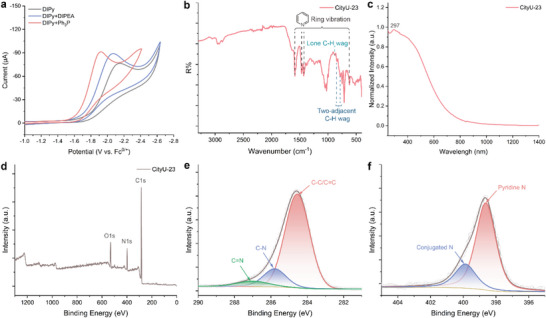
The investigation of applied potential and the structural characterization of **CityU‐23** thin film. a) Cyclic voltammetry of DIPy with (or without) DIPEA and Ph_3_P. b) FTIR spectrum, c) UV–vis spectrum, d) XPS full survey, e) high‐resolution C1s spectra, and f) high‐resolution N1s spectra of **CityU‐23** thin film.

**Scheme** **1** is the structures of two N‐containing poly(*p*‐phenylene)s (**CityU‐23** (poly(2,5‐pyridine)) and **CityU‐24** (poly(2,5‐pyrazine)), which were obtained through cathodic‐dehalogenation polymerization of DIPy and DBPz. To investigate the reduction potential of monomers, DIPy was selected as the example to perform the cyclic voltammetry (**Figure** **1**a; the full spectra can be found in Figure S1, Supporting Information). The onset reduction potential and the reduction peak are at −1.80 and −2.14 V (vs Fc^0/+^), respectively. Interestingly, the onset potential and the reduction peak would synchronously shift ≈0.07 V to the positive potential, and the maximum current tends to be larger after the addition of DIPEA. When the Ph_3_P is added, the onset potential and the reduction peak further positively shift by ≈0.08 V and 0.15 V, respectively, and are accompanied by an increase in maximum current. Therefore, the Ph_3_P can decrease the reduction potential and promote the dehalogenation process. The structure of **CityU‐23** was determined by the Fourier‐transform infrared spectroscopy (FTIR) (Figure 1b). The specific peaks of 1588, 1462, 1432, and 621 cm^−1^ are assigned to the pyridine ring vibration.^[^ ^37^ ^]^ The bands of 789 and 844 cm^−1^ belong to the two adjacent C─H wags in different chemical atmospheres, indicating the formation of **CityU‐23** and probably in mixture form of head‐to‐head, head‐to‐tail, and tail‐to‐tail.^[^ ^37^, ^38^ ^]^ The peak at 883 cm^−1^ can be ascribed to the lone C─H wag near the nitrogen atom of pyridine rings.^[^ ^38^ ^]^ Therefore, the substitution should occur at the 2‐ and 5‐positions previously occupied by the halogen atoms. The UV–vis spectrum shows a broad absorption peak, indicating the successful polymerization (Figure 1c). The X‐ray photoelectron spectroscopy (XPS) survey spectrum only shows signals of C, N, and O without the appearance of iodine, indicating the successful dehalogenation to form **CityU‐23**.(Figure 1d) Furthermore, **CityU‐23** not only shows the C─N (285.8 eV) and C═N (287.1 eV) bonds in high‐resolution C1s spectra (Figure 1e) but also displays the pyridine N (398.6 eV) and conjugated N (399.9 eV) in the high‐resolution N1s spectra (Figure 1f). The ratio of N to C is ≈1:5.36 (the theoretical ratio is 1:5), suggesting the successful polymerization via the electrochemical‐dehalogenation process.^[^ ^4^, ^39^ ^]^

### 2.2 The Morphological Characterization

**Figure 2:**
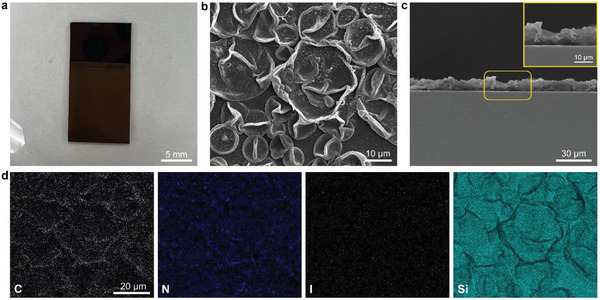
The morphology of **CityU‐23** thin film fabricated. a) The photograph of the **CityU‐23** thin film on a Si substrate. b) The top‐view SEM image of the **CityU‐23** thin film. c) The cross‐section SEM image of the **CityU‐23** thin film. Insert is the enlarged image of the area circled by yellow rounded rectangle frame. d) The corresponding EDX mappings of elements C, N, I, and Si of (b).

The light‐brown **CityU‐23** thin film can be directly fabricated on conductive substrates, for example, silicon (**Figure** **2**a). The micromorphology was observed under a scanning electron microscope (SEM). The **CityU‐23** thin film covers the substrate in a wrinkled form, like a continuous film with deflated bubbles, which differs from homocyclic aromatic polymer films (Figure 2b,c)^[^ ^7^ ^]^. The corresponding EDX mappings also demonstrate the uniformity of **CityU‐23** thin film. The lack of iodine signal in conjunction with the remaining carbon and nitrogen signals echoes the experiment data mentioned above, further demonstrating the completion of the electrochemical‐dehalogenation process. We further observe the morphology of the as‐prepared **CityU‐23** thin film at nanostructure scale via TEM and no crystalline features can be observed (Figure S2, Supporting Information).

### 2.3 The Extension of the Substrates

Since electrons directly serve as the catalyst, we postulate that the heterocyclic aromatic polymer film can be fabricated by different anodes and onto other conductive substrates.^[^ ^7^, ^21^ ^]^ To investigate the effect of the anode, we changed the platinum into zinc (or nickel) in the same size and conducted the polymerization under the same condition. However, except for the thickness, there were almost no changes in the morphology of the **CityU‐23** thin film (Figure S3, Supporting Information). Moreover, the **CityU‐23** thin films were prepared onto different conductive substrates, including gold, nickel, and ITO glass, without significant structural differences, where Pt serves as the anode (Figure S4, Supporting Information). However, the conductance of the substrates will slightly affect the morphology of the **CityU‐23** thin films. The surface of the thin film becomes smoother with increasing the conductance of the substrates, however, the overall morphology (the films are still in a wrinkled structure) shows no significant change (Figure S6a–c, Supporting Information). The uniformity and the smoothness of the as‐obtained **CityU‐23** thin film are also affected by the conductive substrates themselves. Although the FTO substrate has similar conductance with ITO‐2, the surface of the **CityU‐23** thin film fabricated on the FTO substrate looks rougher than that of ITO‐2 (Figure S6b,e, Supporting Information). Moreover, the **CityU‐23** thin film on the Si is the most uniform and smoothest (Figure S6b,d,e, Supporting Information).

### 2.4 The Scope Extension of Cathodic‐Dehalogenation Polymerization

The successful fabrication of **CityU‐23** thin film inspired us to extend the scope of precursors to multi‐heteroatoms substituted aromatic compounds, for example, DBPz (Scheme 1b). Interestingly, the DBPz shows two irreversible peaks during the reduction process. We hypothesize that it may undergo two steps of the dehalogenation process, though theoretically, the two bromine groups on the pyrazine rings are in the same chemical atmosphere. However, the reaction mechanism is still unclear and needs further investigation. The onset potentials of the DBPz were −1.68 and −1.79 V (vs Fc^0/+^) after the addition of Ph_3_P and DIPEA, respectively, which positively shifted ≈0.13 V (for Ph_3_P) and ≈0.02 V (for DIPEA) from the pristine (−1.81 V vs Fc^0/+^) (Figure S7a, Supporting Information). The reduction peaks also show a similar change after the addition of Ph_3_P and DIEPA. Although the addition of Ph_3_P could lower the absolute value of the DBPz reduction potential and significantly increase the first reduction peak current, the second reduction peak current is decreased, indicating the probable inhibition of the second‐step dehalogenation. The as‐obtained **CityU‐24** thin film was first characterized by FTIR spectroscopy and shows the vibration bands of pyrazine ring at 1618, 1520, 1472, 1435, 1399, 1156, 1019, and 410 cm^−1^, as well as the fingerprint vibration of lone C─H wag on the pyrazine loacting at 851 cm^−1^, indicating the formation of **CityU‐24** thin film (Figure S7b, Supporting Information).^[^ ^40^, ^41^ ^]^ The UV–vis spectrum shows a broad absorption peak similar to the literature report, indicating the polymerization (Figure S7c, Supporting Information).^[^ ^27^, ^40^ ^]^ The **CityU‐24** thin film was further characterized by XPS. The strong C and N signals and the vanishing of the Br signal indicate the successful dehalogenated polymerization (Figure S7d, Supporting Information). The high‐resolution XPS spectra of C1s and N1s were also conducted (Figure S7e,f, Supporting Information). It shows two peaks with the binding energy of 284.6 and 286.7 eV, corresponding to the C─C/C═C and pyrazine C and N bonds in a conjugated atmosphere, respectively. The single peak at 398.6 eV also represents the same chemical atmosphere of N in the pyrazine ring.^[^ ^42^, ^43^ ^]^ Also, the XPS elemental analysis shows that the ratio of N to C is ≈1:2.27 (theoretical ratio is 1:2). The micromorphology was observed under SEM, showing a continuous and uniform structure different from the **CityU‐23** thin film (Figure S8, Supporting Information). It may result from the symmetry of pyrazine rings and no random polymerization compared with the pyridine rings. Also, the **CityU‐24** thin film shows an amorphous structure at nanostructure scale (Figure S9, Supporting Information). Additionally, the **CityU‐24** thin film can be prepared onto various conductive substrates (i.e., gold, silicon, and ITO glass) (Figure S10, Supporting Information). Similarly, increasing the conductance of the substrates will result in smoother surface of the as‐obtained thin films. The uniformity and smooth of the as‐prepared thin films are also affected by the substrates themselves. However, the overall morphology does not show any significant changes (Figures S5 and S11, Supporting Information).

However, this electron‐assisted dehalogenation polymerization strategy cannot be extended to the heterocyclic compounds with multi‐nitrogen atoms linked together, such as the 3,6‐diiodopyridazine and 3,6‐dibromo‐1,2,4,5‐tetrazine. Perhaps the ‐N═N‐ double bond is more electrochemically active than the carbon‐halogen bonds. Under the cathodic bias, the ‐N═N‐ would competitively undergo a reduction reaction and form ‐NH─NH‐ species. For 3,6‐diiodopyridazine, the onset potentials are −1.53 V (pristine) and −1.526 V (after the addition of DIPEA), and there is not any change in the reduction peak of the pristine and after the addition of DIPEA (Figure S12, Supporting Information). However, the onset potential and the reduction peak shift negatively after the addition of Ph_3_P, though the Ph_3_P can lower the energy requirement for breaking C‐halogen bonds. As to the 3,6‐dibromo‐1,2,4,5‐tetrazine, the electrochemical process is quite complicated (Figure S13, Supporting Information), but it shows a quasi‐reversible reaction with a pair of redox peaks at −0.71 and −0.62 V (vs Fc^0/+^) which is probably assigned to the strong electron‐deficient tetrazine ring because of the decrease in the intensity after the addition of DIPEA.

### 2.5 The HER Performance of **CityU‐23** and **CityU‐24**

**Figure 3:**
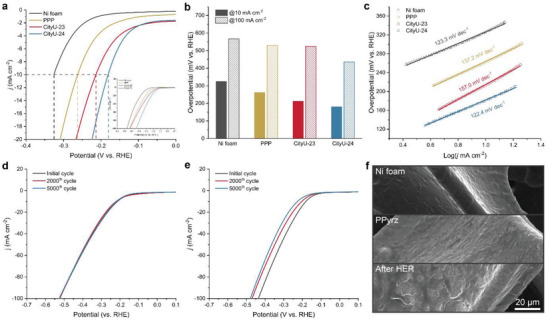
a) The HER polarization curves for PPP, **CityU‐23** and **CityU‐24** thin film on nickel foam (electrolyte: 1 m KOH, scan rate: 10 mV s^−1^). b) Overpotential at 10 and 100 mA cm^−2^. c) Tafel slopes. d) Electrochemical ADT test of **CityU‐23** thin film in 1 m KOH. e) Electrochemical ADT test of **CityU‐24** thin film in 1 m KOH. f) The micromorphology of **CityU‐24** thin film before and after 2000 cycles ADT test.

**Figure 4:**
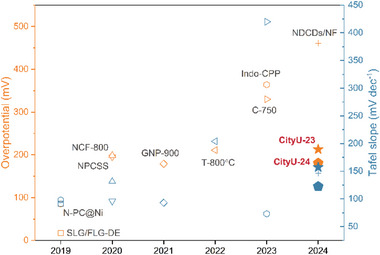
The summary of the metal‐free catalyst HER performance in 1 m KOH in recent five years (selected). Orange color: overpotential; Blue color: corresponding Tafel slope. SLGFLG‐DE: single‐/few‐layer graphene flakes with defective edges;^[^ ^46^ ^]^ N‐PC@Ni: N‐doped porous carbon at Ni foam;^[^ ^47^ ^]^ NPCSS: N‐doped few‐layer porous carbon nanosheets;^[^ ^48^ ^]^ NCF‐800: N‐doped carbon fibers derived from the carbonization of the electron‐spun polyacrylonitrile membrane at 800 °C;^[^ ^49^ ^]^ GNP‐900: a porous N, P, and O‐doped carbon‐based nanosheet;^[^ ^50^ ^]^ T‐800 °C: Tamarind carbonizations – activated carbons under 800 °C;^[^ ^51^ ^]^ C‐750: 2D mesoporous N‐doped activated carbon sheets from the *Acorus Calamus* plant “root” part and pyrolyzed at 750 °C;^[^ ^52^ ^]^ NDCDs/NF: nitrogen‐doped carbon dots from *Luffa acutangula*.^[^ ^53^ ^]^

The HER, as a fundamental step of splitting water into usable hydrogen without any pollutants, always requires a reliable catalyst to achieve economic and highly efficient production for practical applications.^[^ ^28^, ^44^, ^45^ ^]^ Herein, the metal‐free HER catalysis performance of the **CityU‐23** and **CityU‐24** thin films was investigated in 1.0 m of KOH aqueous solution. Compared with the poly(*p*‐phenylene) (PPP) (262.76 and 529.76 mV of overpotential at 10 and 100 mA cm^−2^, respectively) fabricated from the 1,4‐diiodobenzene, the N‐containing aromatic polymers show good alkaline HER catalysis performance. The **CityU‐24** thin film exhibits better HER catalytic performance with small overpotentials of 180.7 and 435.8 mV at the current densities of 10 and 100 mA cm^−2^, respectively, than those of **CityU‐23** thin film with 212.8 mV of overpotential at 10 mA cm^−2^ and 523.8 mV at 100 mA cm^−2^, respectively (**Figure** **3**a,b). The **CityU‐24** thin film shows a Tafel slope of 122.4 mV dec^−1^ lower than that of **CityU‐23** thin film (157 mV dec^−1^), indicating the **CityU‐24** thin film could promote the HER reaction efficiently (Figure 3c). Moreover, the electrochemical impedance spectroscopy (EIS) displays fairly small solution resistance and facile kinetics with mass transfer being the dominant process, which is probably due to the special geometric structure of the Ni foam (Figure S14, Supporting Information). The diffusion of charges within the films gives a Warburg impedance with a unity slope. Further decreasing frequencies, the finite thickness of the film in conjunction with the blocking properties of the impermeable interface result in capacitive behaviors. In addition, among PPP, CityU‐23, and CityU‐24, the PPP film exhibits the slowest diffusion rate, while CityU‐24 shows the fastest diffusion rate. Therefore, replacing the sp^2^ C with more active sp^2^ N in the aromatic rings could bring the polymer alkaline HER catalytic properties, and the HER overpotential decreases with the increasing content of sp^2^ N in the aromatic rings. However, the **CityU‐24** thin film is less stable than the **CityU‐23** thin film during the alkaline HER process (Figure 3d,e). After 2000‐cycle accelerated durability tests (ADT), the surface of **CityU‐24** thin film becomes rough, indicating some damage occurs on its surface and thus decreases the alkaline HER performance (Figure 3f). Although **CityU‐24** shows better catalytic performance for alkaline HER, the multi‐nitrogen introduction into an aromatic ring reduces its stability, thereby further reducing the stability of the polymer during the electrocatalytic process. The alkaline HER performance of these two metal‐free polymers is summarized and shown in **Figure** **4** (for more details, please refer to Table S1, Supporting Information). Among them, the single‐/few‐layer graphene with defective edges (SLG/FLG‐DE) has shown the best performance in metal‐free alkaline HER. After reviewing metal‐free alkaline HER, defect engineering seems to be a predominant trend for improving the HER performance since it could modify the electronic structure, directly serve as active sites, and make active sites more accessible. Our research can be an alternative strategy to create defects in benzene/polycyclic aromatic hydrocarbons by cathodic‐dehalogenation polymerization of nitrogen‐containing monomers.

## 3 Conclusion

The scope of cathodic‐dehalogenation coupling between aromatic compounds has been extended to the N‐containing aromatic compounds (i.e., DIPy and DBPz) to construct two conjugated polymer films (**CityU‐23** and **CityU‐24**) on various conductive substrates. Both metal‐free thin films of CityU‐23 and CityU‐24 show good alkaline HER performance with small overpotentials of 212.8 and 180.7 mV at 10 mA cm^−2^, respectively. The Tafel slopes of CityU‐23 and CityU‐24 thin films were 157 and 122.4 mV dec^−1^, respectively. We found that the increased number of the introduced sp^2^ nitrogen atoms could provide more active sites with better performance in the alkaline HER process. However, more N atoms would inevitably sacrifice the stability. Our research would provide an alternative sight in preparing metal‐free catalysts for HER in alkaline media.

## Conflict of Interest

The authors declare no conflict of interest.

## Supporting information

Supporting Information

## Data Availability

The data that support the findings of this study are available in the supplementary material of this article.
