# Supplementary material for: Constructing N‐Containing Poly(p‐Phenylene) (PPP) Films Through A Cathodic‐Dehalogenation Polymerization Method
Source: Small Methods. 2024 Apr 15;8(11):2400185. doi: 10.1002/smtd.202400185 (PMC11579557; doi:10.1002/smtd.202400185)
Supplement: Supplementary file 1 — Supporting Information [file SMTD-8-2400185-s001.pdf]

small methods

Supporting Information

for *Small Methods*, DOI 10.1002/smtd.202400185

Constructing N-Containing Poly(*p*-Phenylene) (PPP) Films Through A Cathodic-Dehalogenation Polymerization Method

*Xiang Wang, Lei Zhang, Jinghang Wu, Miaomiao Xue, Qianfeng Gu, Junlei Qi, Fangyuan Kang, Qiyuan He, Xiaoyan Zhong and Qichun Zhang**

1. Materials

2,5-diiodopyridine (95%, Sigma-Aldrich), 2,5-dibromopyrazine (>98%, GC, TCI (Shanghai) Development Co., Ltd.), acetonitrile (99.9%, J&K Scientific Ltd), dimethyl sulfoxide (DMSO, 99.8%, J&K Scientific Ltd.), dichloromethane (DCM, ACS grade, Anaqua Global International Inc. Ltd.), ethanol (EtOH, ACS grade, Anaqua Global International Inc. Ltd.), tetrabutylammonium tetrafluoroborate (98%, $TBABF_4$, Energy Chemical), N,N-diisopropylethylamine (DIPEA, 99.5%, J&K Scientific Ltd.), triphenylphosphine (Ph3P, >95%, GC, Sigma-Aldrich), 3 Å molecular sieves (Shanghai Aladdin Bio-Chem Technology Co., Ltd.). All the chemicals and reagents are directly used without further purification unless otherwise noted. Deionized (DI) water was used throughout the experiment.

The 3 Å molecular sieves were completely activated at 300°C in vacuum furnace for 3 hours and stored in glove box. The anhydrous MeCN and DMSO were obtained by drying over the post-activated 3 Å molecular sieve\s in glove box several times for at least 24 hours each time. The electrolyte $TBABF_4$ was purified by recrystallization from absolute EtOH three times, dried in a vacuum oven at 80°C for approximate 24 hours, and stored in nitrogen filled glove box.

2. Instruments and measurements

2.1. Fourier-transformed infrared spectroscopy (FTIR)

The FTIR spectra were obtained on PerkinElmer Spectrum II with attenuated total reflectance (ATR) mode.

2.2. Ultraviolet-visible spectroscopy (UV-Vis)

The UV-Vis apparatus was Hitachi UH4150 UV-VIS-NIR Spectrophotometer. The as-prepared thin film was gently transferred onto an appropriate quartz plate and fixed onto the sample state for the UV-Vis measurement.

2.3. X-ray photoelectron spectroscopy (XPS)

The XPS was conducted on Thermo Fisher ESCALAB XI+ X-ray Photoelectron Spectrometer. The as-prepared thin film was smoothly covered on the Si/SiO_2 substrate and fixed onto the indium sample stage with double-sized tape before the XPS measurement. The ratio of N to C was obtained via the elemental analysis module in Thermo Scientific™ Advantage software.

2.4. Scanning electron microscope (SEM) and energy-dispersive X-ray spectroscopy (EDX)

The SEM images were obtained by Thermo Fisher Quattro S Environmental SEM equipped with EDX module. The as-prepared thin film was smoothly covered on the Si/SiO2 substrate and fixed on the sample state with conductive carbon tape. Then the silica wafer with the sample was coated with a thin layer of gold (~10 nm for the cross-section image and ~20 nm for the top-view image). The measurement was carried with an accelerating voltage of 15 kV.

2.5. Transmission electron microscope (TEM)

The TEM images were obtained under JEM-ARM300F2 with 80 kV of accelerating voltage.

2.6. Electrochemical analysis

The cyclic voltammetry (CV) was conducted on CH Instruments 760E electrochemical workstation. The procedure and the device preparation are similar to the previous report.[1] All measurements were carried at a scan rate of 50 mV/s in MeCN/DMSO (v/v = 3:7) mixture containing 0.1 M of $TBABF_4$ as the supporting electrolyte.

In the hydrogen evolution reaction (HER) measurement, a standard three-electrode system was employed, consisting of nickel foam coated with CityU-23 (or CityU-24) thin film as the working electrode, a saturated Ag/AgCl electrode as the reference electrode, and carbon rod as the counter electrode. The electrolyte was 1 M KOH aqueous solution. All the potentials reported in this work were converted to reversible hydrogen electrode (RHE) via the equation:

$$E(vs.RHE) = E(vs.Ag/AgCl) + E_{Ag/AgCl} = E(vs.Ag/AgCl) + (0.197\ V) + 0.0592pH$$
$$= E(vs.Ag/AgCl) + 1.0258V$$

For electrochemical measurements, fast cyclic voltammograms (400 cycles, 100 mV s^{-1}, from 0.4258~0.6258 V (vs. RHE)) were conducted to activate the catalysts. The linear sweep voltammetry (LSV) curves were obtained at a scan rate of 10 mV s^{-1} in N_2-saturated solution under 800 rpm without iR compensation. The accelerated durability tests (ADT) were conducted via cyclic voltammograms in the range of -0.2742~0.1258 V (vs. RHE) at a scan rate of 100 mV s^{-1}.

The electrochemical impedance spectroscopy (EIS) was obtained under the open circuit voltage with 5 mV of perturbation in a 1 M of KOH aqueous solution. The frequencies range from 1 Hz to 1MHz.

The I-V curve was obtained via a precision source measurement unit (Keysight B2902a) equipped with a probe station. The probes are 0.5 mm in diameter and 1 cm apart.

3. The general procedure for the polymerization and corresponding thin film fabrication

3.1. The fabrication of CityU-23 (poly(2,5-pyridine) thin film

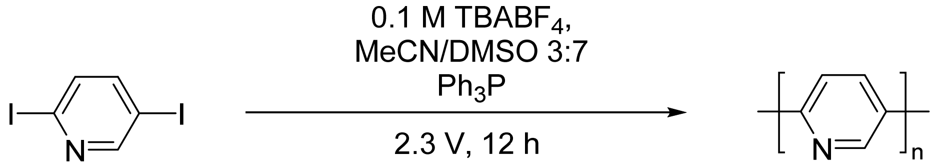

In general, a divided electrolyte cell equipped with a glass sand core (or 0.45μm filter membrane) was used in the fabrication process. The anodic solution was prepared by dissolving $TBABF_4$ (329.27 mg, 1 mmol) in 10 mL of anhydrous DMSO/MeCN (v/v = 7:3) mixture. 2,5-Diiodopyridine (33 mg, 0.0997 mmol, DIPy), triphenylphosphine (157 mg, 0.5986 mmol, 6 equiv.) [or N,N-diisopropylethylamine (104.5 μL, 0.6 mmol, 6.02 equiv.)], and $TBABF_4$ (329.27 mg, 1 mmol) were dissolved in 10 mL of anhydrous DMSO/MeCN (v/v = 7:3) mixture to obtain the cathodic reaction solution. Then, the anodic and cathodic reaction solutions were separately added into a flame-dried divided cell equipped with Pt anode and silicon cathode through a 0.45 μm PTFE filter. After that, the electrolyte cell was sealed and brought out of the glove box for polymerization under the condition of a constant volt at 2.3 V for about 12 hours at room temperature via Matsusada Precision DC Power Supply R4G series. After the polymerization, the cathode was carefully removed from the solution and gently immersed into a sample bottle or a glass petri dish filled with enough THF and cleaned with THF, DCM, EtOH, and a gradient of EtOH/DI water mixture several times. Then, the **CityU-23** thin film was pre-dried in the air before transferring to a vacuum over (100°C) for further drying.

Also, the film can be fabricated via the dynamic cyclic voltammetry method from -0.3 V to -2.3 V (vs. $Fc^{0/+}$) for 20 cycles at the scan rate of 50 mV/s.

3.2. The fabrication of CityU-24 (poly(2,5-pyrazine) thin film

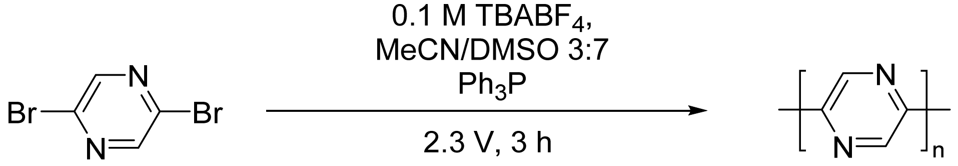

In general, a divided electrolyte cell equipped with a glass sand core (or 0.45μm filter membrane) was used in the fabrication process. The anodic solution was prepared by dissolving $TBABF_4$ (329.27 mg, 1 mmol) in 10 mL of anhydrous DMSO/MeCN (v/v = 7:3) mixture. 2,5-dibromopyrazine (46 mg, 0.193 mmol, DBPz), triphenylphosphine (159 mg, 0.606 mmol, 3.13 equiv.) [or N,N-diisopropylethylamine (104.5 μL, 0.6 mmol, 3.10 equiv.)], and $TBABF_4$ (329.27 mg, 1 mmol) were dissolved in 10 mL of anhydrous DMSO/MeCN (v/v = 7:3) mixture to obtain the cathodic reaction solution. Then, the anodic and cathodic reaction solutions were separately added into a flame-dried divided cell equipped with Pt anode and silicon cathode through a 0.45 μm PTFE filter. After that, the electrolyte cell was sealed and brought out of the glove box for polymerization under the condition of a constant volt at 2.3 V for about 3 hours at room temperature via Matsusada Precision DC Power Supply R4G series. After the polymerization, the cathode was carefully removed from the solution and gently immersed into a sample bottle or a glass petri dish filled with enough THF and cleaned with THF, DCM, EtOH, and a gradient of EtOH/DI water mixture several times. Then, the **CityU-24** thin film was pre-dried in the air before transferring to a vacuum over (100°C) for further drying.

Also, the film can be fabricated via the dynamic cyclic voltammetry method from -0.3 V to -2.3 V (vs. $Fc^{0/+}$) for 20 cycles at the scan rate of 50 mV/s.

4. The full cyclic voltammetry curves of DIPy

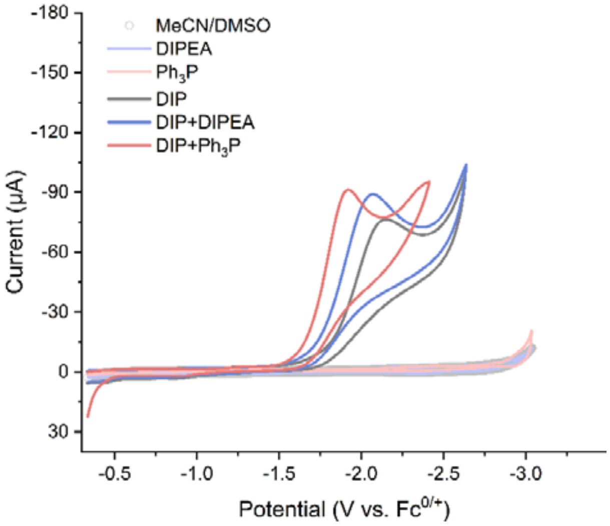

Figure S1. The full cyclic voltammetry of DIPy with (or without) DIPEA and Ph_3P.

5. The bright-field TEM image of CityU-23

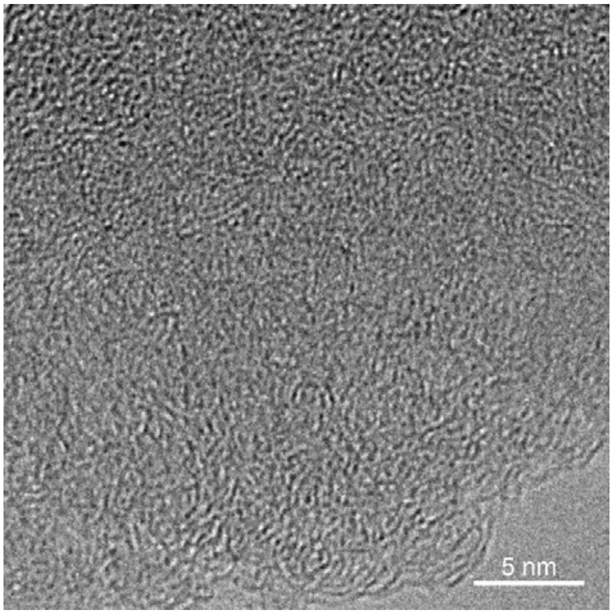

Figure S2. The bright-field TEM image of CityU-23 thin film.

6. The morphology of CityU-23 thin film fabricated by different anodes

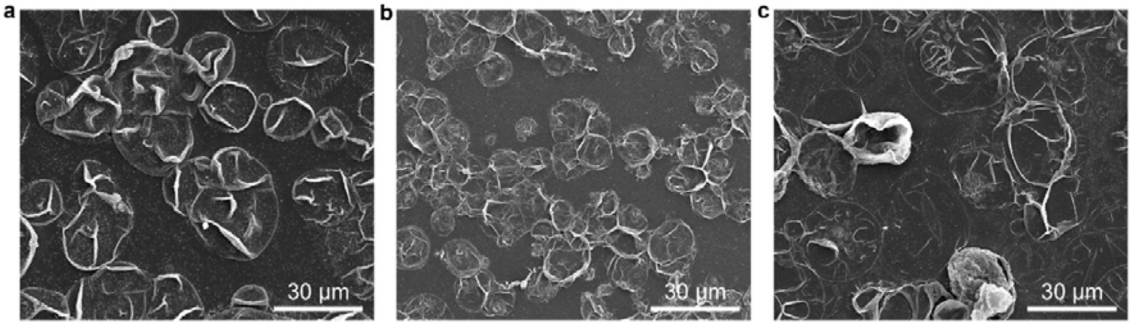

Figure S3. The morphology of the **CityU-23** thin films fabricated by different anodes. a) Pt as the anode. b) Zn as the anode. c) Ni as the anode.

7. The FTIR spectra of CityU-23 thin film fabricated on different conductive substrates

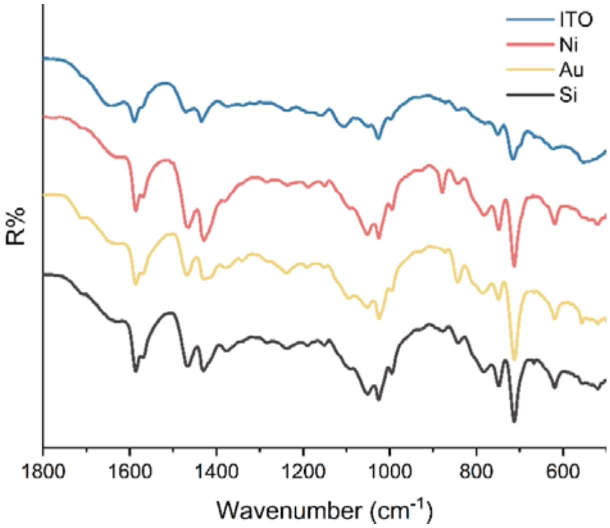

Figure S4. The FTIR spectra of **CityU-23** thin films fabricated on different conductive substrates.

8. The morphology of CityU-23 thin film fabricated on different conductive substrates

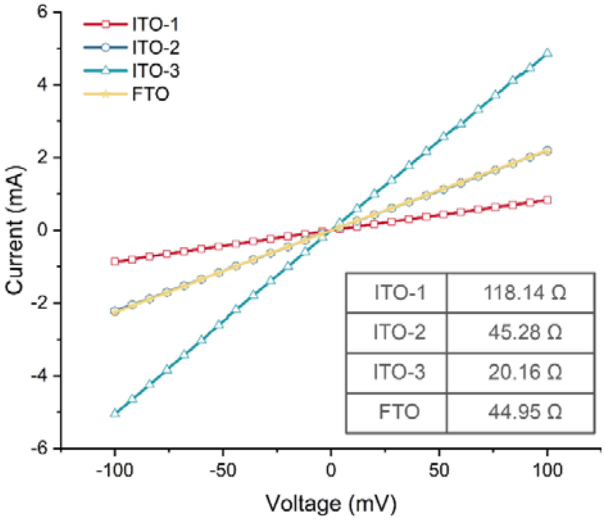

Figure S5. The I-V curve of ITO and FTO glasses.

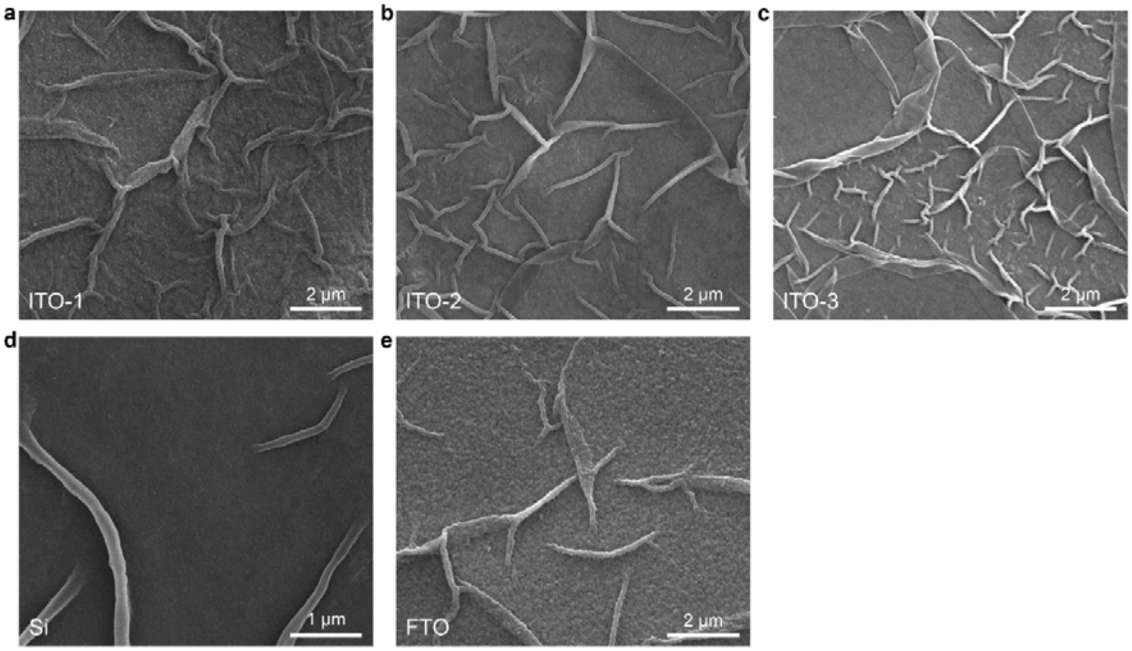

Figure S6. The morphology of CityU-23 thin film fabricated on different conductive substrates. a) ITO-1, b) ITO-2, c) ITO-3, d) Si, and e) FTO.

9. The scope extension

9.1. CityU-24

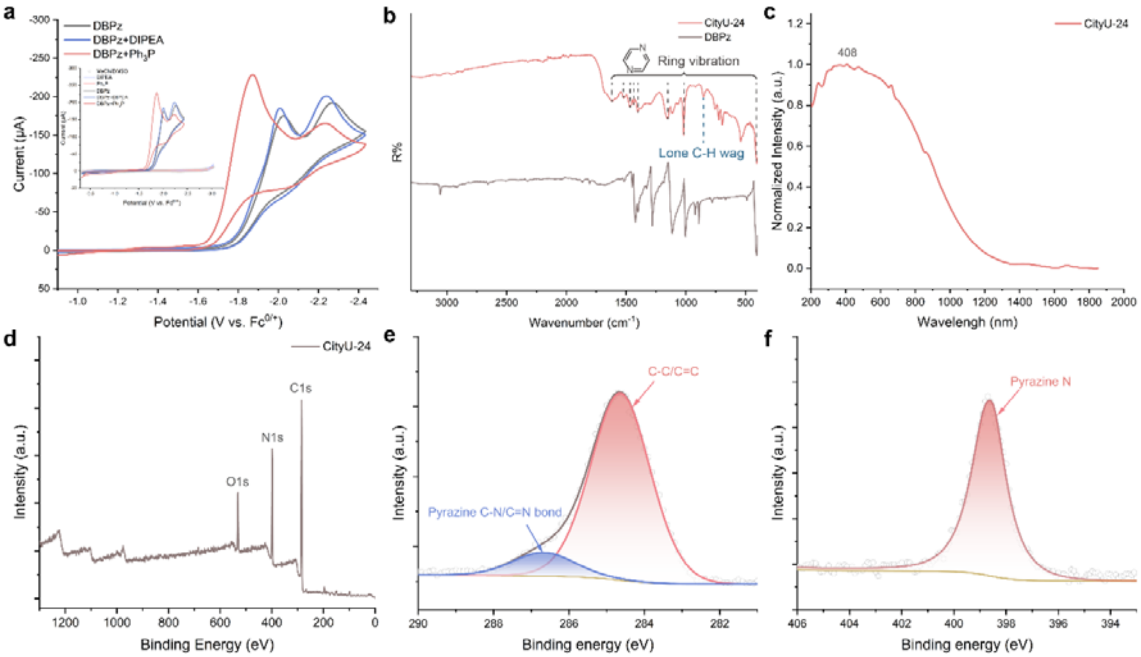

Figure S7. The investigation of applied potential and the structural characterization of **CityU-24** thin film. a) Cyclic voltammetry of DBPz with or without DIPEA and Ph_3P. Insert is the full CV curves. b) FTIR spectra of **CityU-24** thin film and DBPz monomer. c) UV-Vis

spectrum, d) XPS full survey, e) high-resolution C1s spectra, and f) high-resolution N1s spectra of **CityU-24** thin film.

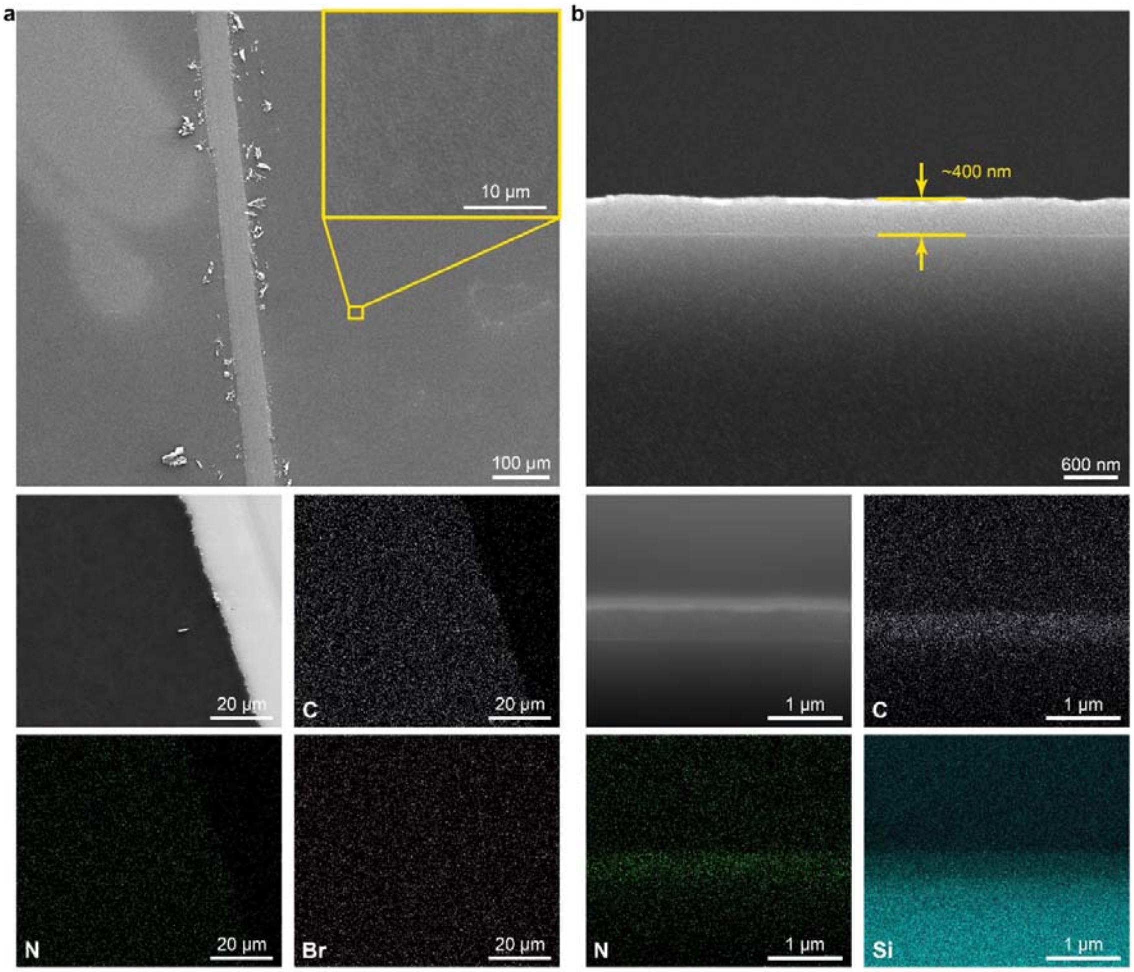

Figure S8. The SEM images of the **CityU-24** thin film on a silicon substrate. a) The top view of the CityU-24 thin film with artificial scratch and EDX mappings of elements C, N, and Br. Insert is an enlarged SEM image of a yellow rectangular circled area. b) The cross-section of the **CityU-24** thin film and EDX mappings of elements C, N, and Si.

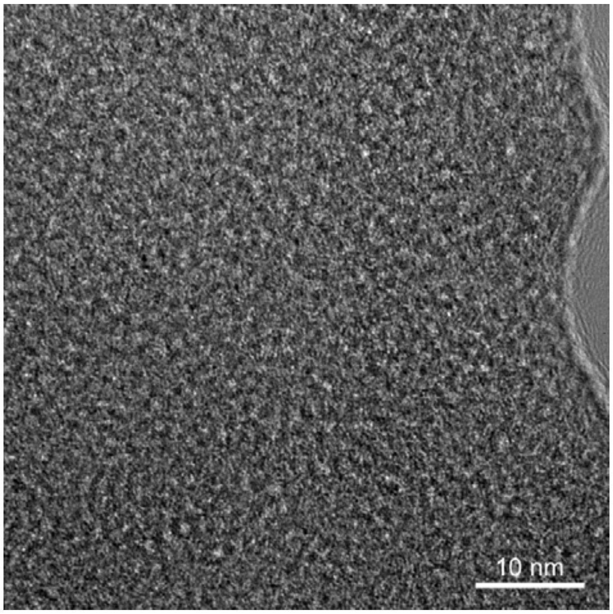

Figure S9. The bright-field TEM image of the CityU-24 thin film.

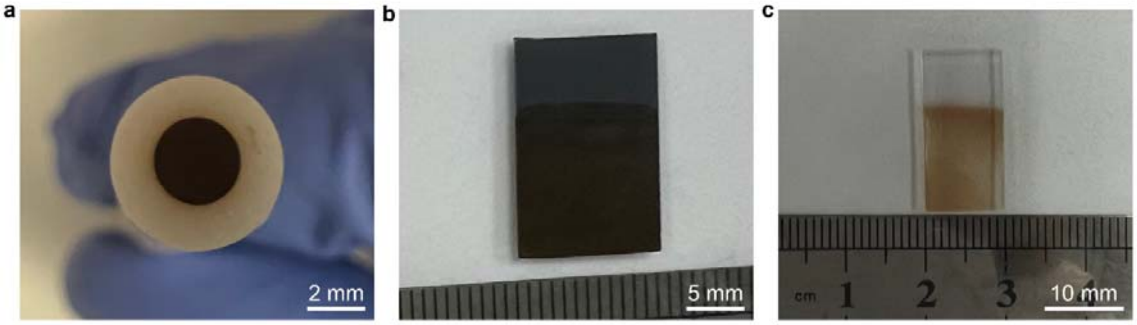

Figure S10. The photographs of the **CityU-24** thin film fabricated on different substrates and in different sizes. a) Gold disk electrode with a diameter of 3 mm. b) Si. c) ITO glass.

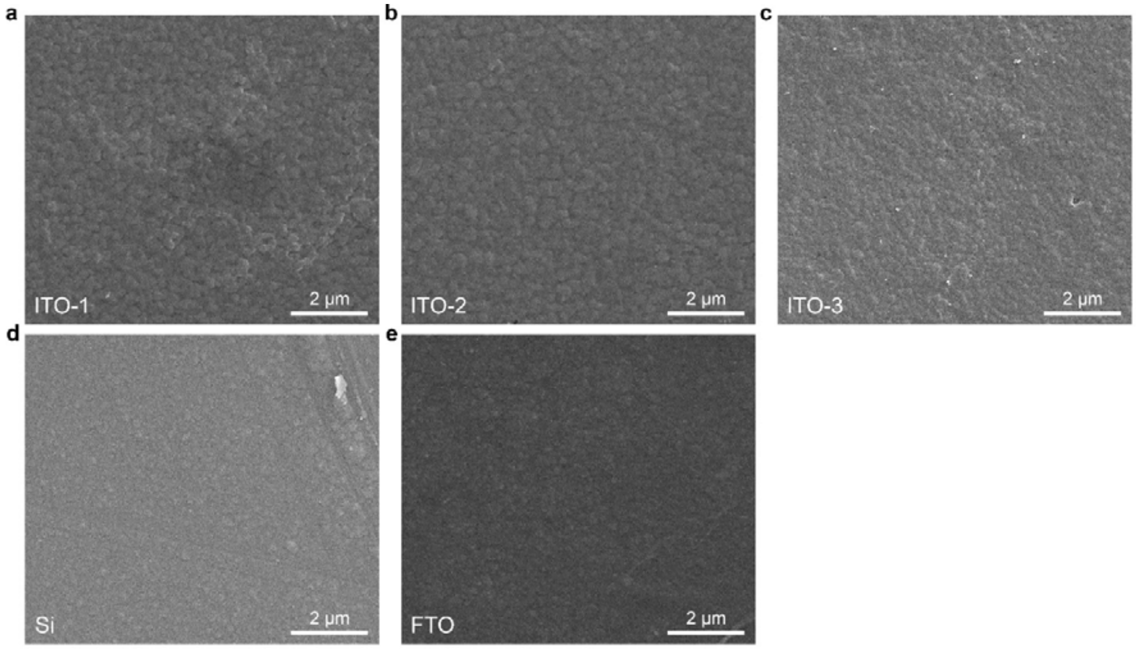

Figure S11. The morphology of CityU-23 thin film fabricated on different conductive substrates under the same reaction conditions. a) ITO-1, b) ITO-2, c) ITO-3, d) Si, and e) FTO.

9.2. Poly(3,6-pyridazine)

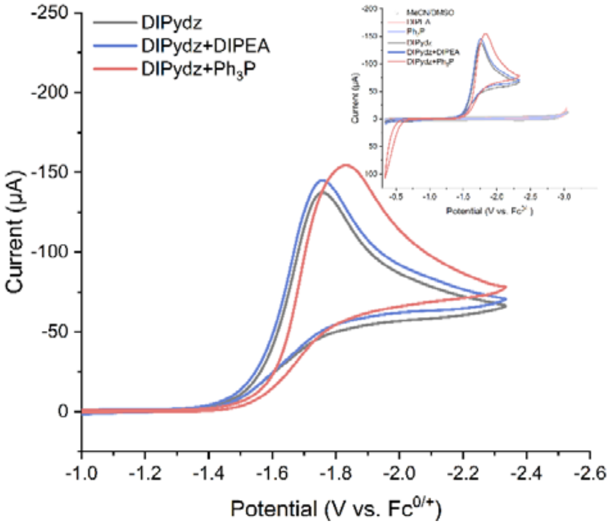

Figure S12. The cyclic voltammetry of 3,6-diiodopyridazine.

9.3. Poly(3,6-tetrazine)

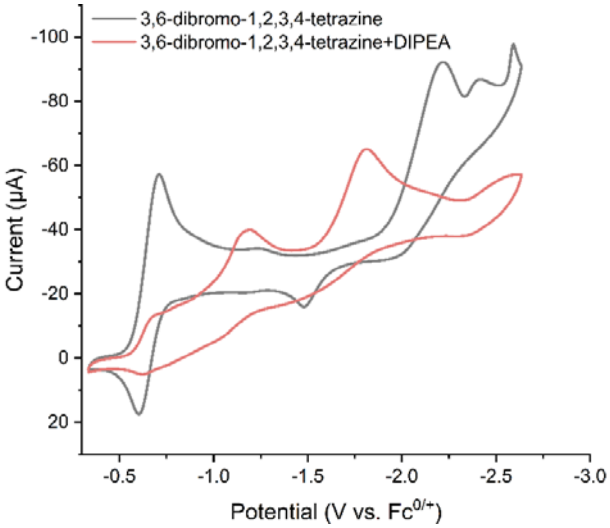

Figure S13. The cyclic voltammetry of 3,6-dibromo-1,2,4,5-tetrazine.

10. The EIS in 1 M of KOH aqueous solution

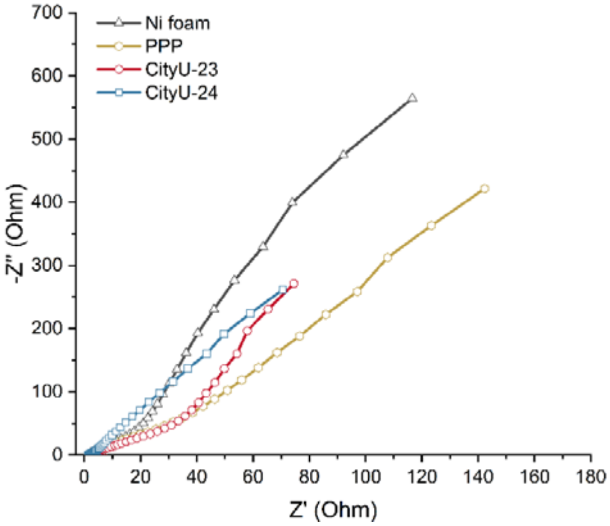

Figure S14. The EIS curves of as-prepared thin films in 1 M of KOH aqueous solution.

11. The summary of the alkaline HER performance of the catalyst in 1 M of KOH aqueous solution

Table S1. The summary of the alkaline HER performance of the metal-free catalysts in 1 M of KOH aqueous solution.

Catalyst	Overpotential @ 10 mA cm^{-2} [mV]	Tafel slope [mV dec^{-1}]	Published year	Reference
S-C3N4-CNT-CF	131	79	2016	[2]
ONPPGC/OCC	446	154	2016	[3]

N-GMT	432	N.A.	2016	[4]
NS-CNT	400 (@ 5 mA cm^{-2})	133	2016	[5]
N,S-CN	380	103	2017	[6]
N,P-CN	490	118	2017	[6]
N-CN	570	97	2017	[6]
N,B-CN	730	87	2017	[6]
HPNS	330	63	2019	[7]
SLG/FLG-DE	85	91	2019	[8]
PC@Ni	200	109	2019	[9]
N-PC@Ni	179	98	2019	[9]
NPCSS	195	96	2020	[10]
NCF-700	240.3	178.1	2020	[11]
NCF-800	198.6	131.3	2020	[11]
NCF-900	283.5	208.2	2020	[11]
GNP-900	179	93	2021	[12]
NP-900	288	104	2021	[12]
GP-900	315	139	2021	[12]
GN-900	370	168	2021	[12]
GNP-700	322	161	2021	[12]
GNP-500	429	180	2021	[12]
T-700℃	230	229	2022	[13]
T-800℃	221	204	2022	[13]
T-900℃	269	309	2022	[13]
C-450	570	1373	2023	[14]
C-750	330	420	2023	[14]
Indo-CPP	364	73	2023	[15]
NDCDs/NF	461	146	2024	[16]
CityU-23	212.8	157.0	2024	This work
CityU-24	180.7	122.4	2024	This work

12. References

[1] X. Wang, J. Wu, H. Liu, F. Kang, F. Yan, Q. Zhang, *Macromolecules* **2023**, *56*, 10198–10205.

[2] Z. Peng, S. Yang, D. Jia, P. Da, P. He, A. M. Al-Enizi, G. Ding, X. Xie, G. Zheng, *J. Mater. Chem. A* **2016**, *4*, 12878–12883.

[3] J. Lai, S. Li, F. Wu, M. Saqib, R. Luque, G. Xu, *Energ. Environ. Sci.* **2016**, *9*, 1210–1214.

[4] B. Zhang, H.-H. Wang, H. Su, L.-B. Lv, T.-J. Zhao, J.-M. Ge, X. Wei, K.-X. Wang, X.-H. Li, J.-S. Chen, *Nano Res.* **2016**, *9*, 2606–2615.

[5] K. Qu, Y. Zheng, Y. Jiao, X. Zhang, S. Dai, S. Qiao, *Adv. Energy Mater.* **2017**, *7*, 1602068.

[6] K. Qu, Y. Zheng, X. Zhang, K. Davey, S. Dai, S. Z. Qiao, *ACS Nano* **2017**, *11*, 7293–7300.

[7] N. Prabu, R. S. A. Saravanan, T. Kesavan, G. Maduraiveeran, M. Sasidharan, *Carbon* **2019**, *152*, 188–197.

[8] L. Najafi, S. Bellani, R. Oropesa-Nuñez, B. Martín-García, M. Prato, F. Bonaccorso, *ACS Appl. Energy Mater.* **2019**, *2*, 5373–5379.

[9] C. Sathiskumar, S. Ramakrishnan, M. Vinothkannan, A. Rhan Kim, S. Karthikeyan, D. J. Yoo, *Nanomaterials* **2019**, *10*, 76.

[10] Z. Liu, Q. Zhou, B. Zhao, S. Li, Y. Xiong, W. Xu, *Fuel* **2020**, *280*, 118567.

[11] J. Sun, Q. Ge, L. Guo, Z. Yang, *Int. J. Hydrogen Energ.* **2020**, *45*, 4035–4042.

[12] Z. Liu, M. Wang, X. Luo, S. Li, S. Li, Q. Zhou, W. Xu, R. Wu, *Appl. Surf. Sci.* **2021**, *544*, 148912.

[13] V. Thirumal, R. Yuvakkumar, B. Saravanakumar, G. Ravi, M. Isacfranklin, M. Shobana, A. G. Al-Sehemi, D. Velauthapillai, *Fuel* **2022**, *324*, 124466.

[14] N. Kumaresan, P. Karuppasamy, M. P. Kumar, S. G. Peera, M. S. AlSalhi, S. Devanesan, R. V. Mangalaraja, P. Ramasamy, T. F. De Oliveira, G. Murugadoss, *Mol. Catal.* **2023**, *539*, 113043.

[15] I. Nath, J. Chakraborty, R. Lips, S. Dekyvere, J. Min, R. S. Varma, F. Verpoort, *J. Mater. Chem. A* **2023**, *11*, 10699–10709.

[16] C. Pitchai, S. M. Gopalakrishnan, C.-M. Chen, *Energy Fuels* **2024**, *38*, 2235–2247.
